# A deletion and a duplication in distal 22q11.2 deletion syndrome region. Clinical implications and review

**DOI:** 10.1186/1471-2350-10-48

**Published:** 2009-06-02

**Authors:** Luis Fernández, Julián Nevado, Fernando Santos, Damià Heine-Suñer, Victor Martinez-Glez, Sixto García-Miñaur, Rebeca Palomo, Alicia Delicado, Isidora López Pajares, María Palomares, Luis García-Guereta, Eva Valverde, Federico Hawkins, Pablo Lapunzina

**Affiliations:** 1 Instituto de Genética Médica y Molecular (INGEMM), Hospital Universitario La Paz, Paseo de la Castellana 261, 28046 Madrid, Spain; 2 CIBER de Enfermedades Raras (CIBERER), Madrid, Spain; 3 Secció de Genètica, Hospital Universitari Son Dureta, Palma de Mallorca, Spain; 4 Servicio de Cardiología Infantil, Hospital Universitario La Paz, Madrid, Spain; 5 Servicio de Neonatología, Hospital Universitario La Paz, Madrid, Spain

## Abstract

**Background:**

Individuals affected with DiGeorge and Velocardiofacial syndromes present with both phenotypic diversity and variable expressivity. The most frequent clinical features include conotruncal congenital heart defects, velopharyngeal insufficiency, hypocalcemia and a characteristic craniofacial dysmorphism. The etiology in most patients is a 3 Mb recurrent deletion in region 22q11.2. However, cases of infrequent deletions and duplications with different sizes and locations have also been reported, generally with a milder, slightly different phenotype for duplications but with no clear genotype-phenotype correlation to date.

**Methods:**

We present a 7 month-old male patient with surgically corrected ASD and multiple VSDs, and dysmorphic facial features not clearly suggestive of 22q11.2 deletion syndrome, and a newborn male infant with cleft lip and palate and upslanting palpebral fissures. Karyotype, FISH, MLPA, microsatellite markers segregation studies and SNP genotyping by array-CGH were performed in both patients and parents.

**Results:**

Karyotype and FISH with probe N25 were normal for both patients. MLPA analysis detected a partial *de novo *1.1 Mb deletion in one patient and a novel partial familial 0.4 Mb duplication in the other. Both of these alterations were located at a distal position within the commonly deleted region in 22q11.2. These rearrangements were confirmed and accurately characterized by microsatellite marker segregation studies and SNP array genotyping.

**Conclusion:**

The phenotypic diversity found for deletions and duplications supports a lack of genotype-phenotype correlation in the vicinity of the LCRC-LCRD interval of the 22q11.2 chromosomal region, whereas the high presence of duplications in normal individuals supports their role as polymorphisms. We suggest that any hypothetical correlation between the clinical phenotype and the size and location of these alterations may be masked by other genetic and/or epigenetic modifying factors.

## Background

DiGeorge syndrome (DGS) and Velocardiofacial syndrome (VCFS) are genetic disorders affecting pharyngeal and neurobehavioural development [1] that result in conotruncal congenital heart defects (CHD), velopharyngeal insufficiency, hypoparathyroidism, thymic aplasia or hypoplasia, craniofacial dysmorphism, learning difficulties and psychiatric disorders [2,3]. Interstitial microdeletions in 22q11.2 have been identified as the underlying cause in most cases of DGS [4], VCFS [5] and apparently isolated conotruncal CHD [6].

Deletions in 22q11.2 cluster into a standard 3 Mb deletion in 87% of the cases, a smaller, proximally nested 1.5 Mb deletion in 7% and other atypical deletions, nested, overlapping or adjacent to the typically deleted region (TDR) [7,8]. By non-allelic homologous recombination (NAHR) after asynchronous replication [9], large low-copy repeats in 22q11.2 (LCR22s A to D) mediate recurrent deletions [7], whereas recently described uncommon deletions are flanked by smaller LCRs (E to H) [10] or alternative breakpoints [5,11-24].

Different point mutations [25,26], balanced translocation breakpoints [27-29] and shortest regions of deletion overlap (SRO) [11-16,30,31] in 22q11.2 have been compared in order to identify candidate genes for the 22q11.2 deletion syndrome phenotype. However, no clear genotype-phenotype correlation has been found [5,32] and identical alterations, even within members of the same family, show high phenotypic diversity and variable expressivity or incomplete penetrance [33-35]. Systematic clinical sorting of patients with non-overlapping deletions has recently shown that an ascertainment bias could be eclipsing different phenotypes or even what would be different syndromes [19,23,36].

22q11.2 duplication syndrome has also been recently characterized as a different clinical entity [37] with features overlapping 22q11.2 deletion syndrome [38]. Since fewer duplications have been reported, it is suspected that the diagnosis of this condition is also biased [39-43], a fact supported by its clinical diversity, generally ranging from a milder, cognitive/behavioral to an apparently normal phenotype in these patients [41,43-48]. This lower severity suggests that duplications with sizes that range from 3 to 6 Mb are less deleterious than deletions [37] and therefore are more likely to be inherited at reduced penetrance [43]. Again, LCRs work as recombination substrates for these rearrangements [37], and different sizes have also been described [43,49].

In this paper we present two patients referred to us for genetic diagnosis of 22q11.2 deletion syndrome. The first patient was found to harbor an atypical deletion and the second one an inherited atypical duplication in the distal segment of the TDR covering LCRs C and D [7]. We discuss screening diagnostic strategies for patients referred for 22q11.2 deletion testing as well as the clinical implications of these findings for a potential genotype-phenotype correlation.

## Methods

Samples and pictures from patients and their families were obtained after informed consent. Ethical approval was obtained for this study from the IRB at Hospital Universitario La Paz in Madrid (HULP-CEIC-PI347). Research was performed in compliance with the Declaration of Helsinki.

### Patient 1

**Figure 1:**
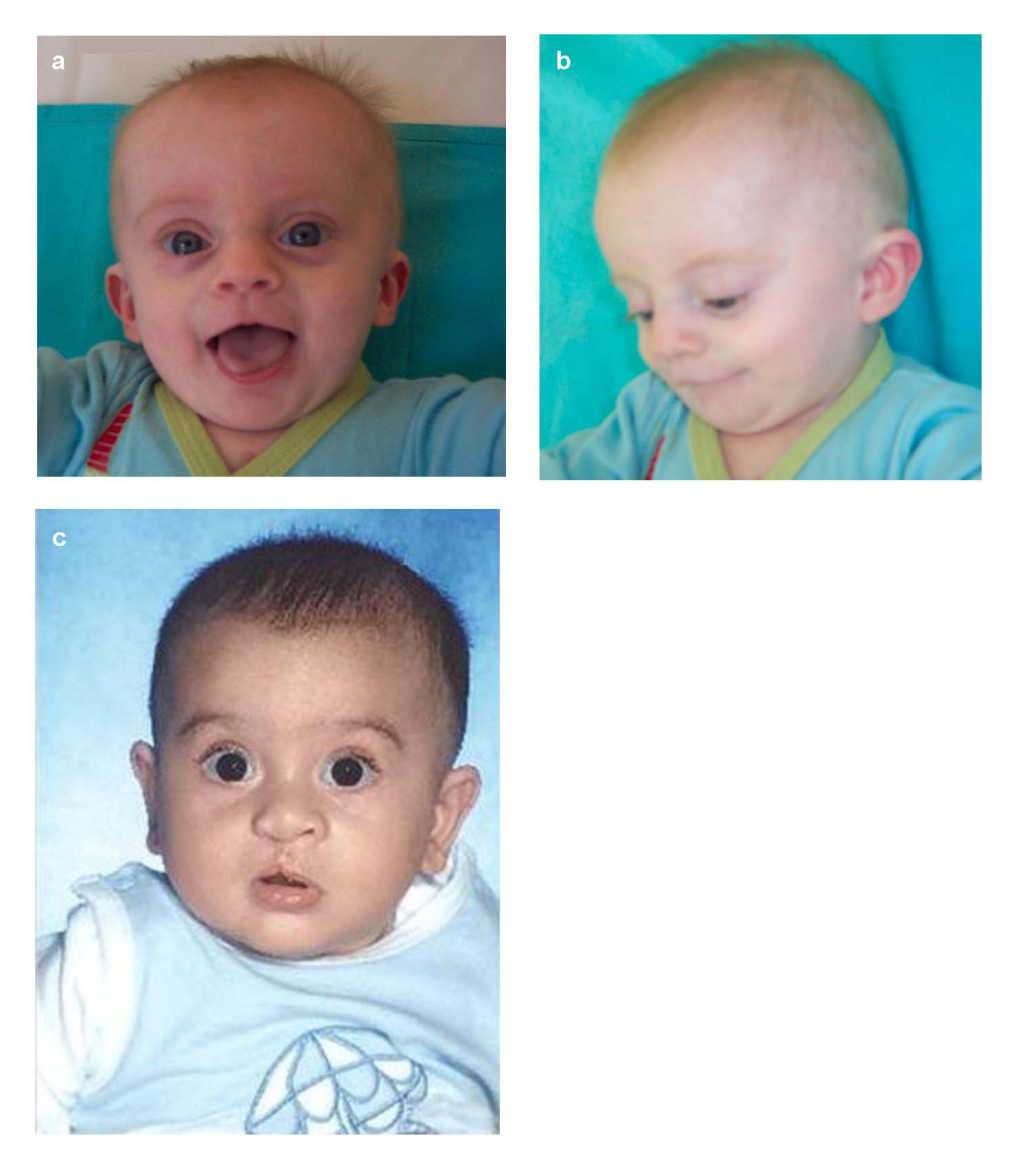
**Facial appearance of Patients 1 and 2**. Patient 1 (a, b); Patient 2 (c).

A male infant was the first child of healthy, non-consanguineous parents of age 39 and 36 years. Due to menstrual dysfunction in the mother, artificial insemination was performed with an oocyte donation from a woman of unknown age. The child was born at 37 weeks of gestation and weighed 2450 g (10th–25th centile). At age 3 months he was referred to Pediatric Cardiology because of respiratory distress and an ASD (ostium secundum type) together with multiple muscular and perimembranous VSDs. He was examined by a clinical geneticist and noted to have some dysmorphic features such as a triangular facies with a slighty small chin and a broad nose (Figure 1a, b). He had a poor head control which he finally achieved at the age of 4 months. Cardiovascular surgery was performed at that time. At age 7 months he was re-evaluated; his growth parameters were: height 62.5 cm (< 5th centile), weight 5540 g (< 3rd centile) and OFC 41 cm (5–10th centile). He was not sitting unsupported yet.

### Patient 2

A male newborn was born to healthy 37 year-old parents after Cesarean section due to lack of progress at 33 weeks of gestation. Birthweight was 2290 g (75th centile). He was referred to the Neonatology intensive care unit because of respiratory distress. He was noted to have a right cleft lip with a complete cleft palate, and upslanting palpebral fissures with no other significant features on examination. He made good progress and was finally discharged two weeks later. He was reviewed in clinic at age five months. His development was within the normal range. His growth parameters were: height 62 cm, weight 6500 g and OFC 42.5 cm (all measurements between the 10th and 25th centiles) (Figure 1c). His cleft lip had been repaired and the cleft palate operation was scheduled for the age of nine months. A later review at age 13 months and a half showed a good progress and a normal development.

### Karyotype and FISH

Cytogenetic analysis was performed after standard harvesting of peripheral blood lymphocytes. Metaphase chromosomes were G-banded. Fluorescence in situ hybridization was performed on peripheral blood lymphocytes, with probe N25 (D22S1660–D22S1646) (Kreatech Diagnostics, Amsterdam, The Netherlands). A minimum of 15 metaphase cells were assessed under a fluorescence microscope (Leica Microsystems, Wetzlar, Germany).

### MLPA

Multiplex ligation-dependent probe amplification was performed on DNA from peripheral blood lymphocytes, extracted with Puregene DNA Isolation Kit (Gentra Systems, Minneapolis, MN). Kits P023B, P250 and P324-A1 for DGS/VCFS/CES (MRC-Holland, Amsterdam, The Netherlands) were used. The three kits test 65 loci on 22q11 (8 on the CES region, 37 within the TDR and 20 adjacent distal to the TDR), 2 on 22q13, 7 on 4q, 1 on 7p, 5 on 8p, 2 on 9q, 9 on 10p, 1 on 10q, 6 on 17p and 2 on 18q, most of them involved in the phenotypes of DGS and VCFS. Data analysis was made against up to 5 control samples using the MRC Coffalyser v8 and v9 software [50] or an in-house Excel spreadsheet.

### Microsatellite segregation studies

Eighteen microsatellite markers (CATCH 10, CATCH 20, CATCH 42, CATCH 41, CATCH 11, D22S311, CATCH 12, CATCH 13, CATCH 14, CATCH 39, D22S1709, CATCH 38, CATCH 37, CATCH 36, CATCH 35, D22S446bis, D22S306, D22S303) spanning the region in the vicinity of LCR22-C and LCR22-D were studied. Markers named CATCH were designed by Torres-Juan *et al *[51].

### Array Hybridization profiling and Data Analysis

DNAs were quantified using PicoGreen (Invitrogen Corporation, Carlsbad, CA) and a genome-wide scan of 620,901 tag SNPs was conducted on the probands, using the Illumina Human610-Quad BeadChip according to the manufacturer's specifications (Illumina, San Diego, CA). GenCall scores < 0.15 at any locus were considered "no calls". Image data was analyzed using the Chromosome Viewer tool contained in Beadstudio 3.2 (Illumina, San Diego, CA). The metric used was the log *R *ratio which is the log (base 2) ratio of the observed normalized *R *value for a SNP divided by the expected normalized *R *value [52]. In addition, an allele frequency analysis was applied for all SNPs. All genomic positions were based upon NCBI Build 36 (dbSNP version 126).

## Results

Karyotype and FISH analysis with probe N25 in both patients and their parents showed normal results.

**Figure 2:**
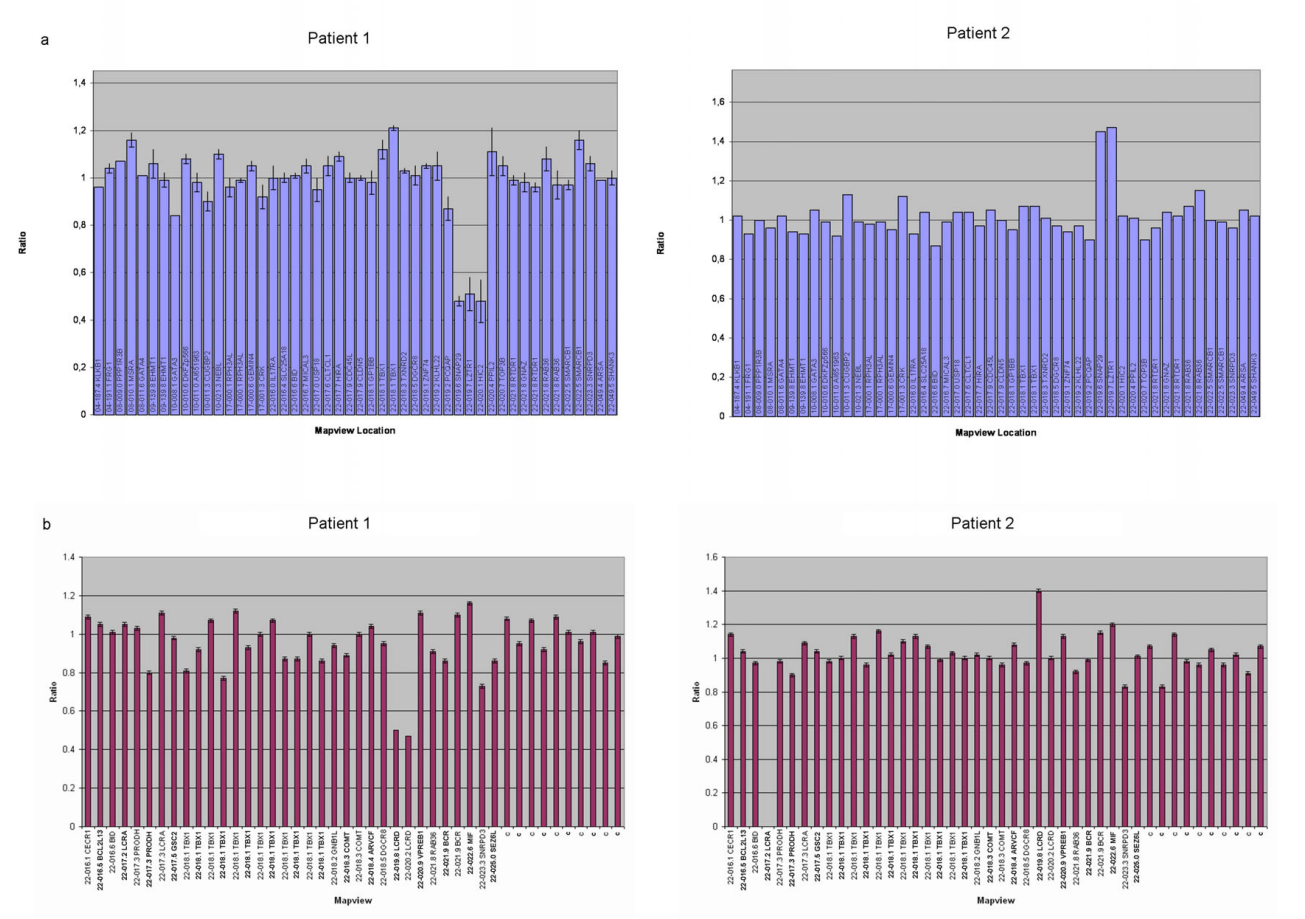
**MLPA results of Patients 1 and 2**. Gene dosage of Patient 1 and Patient 2 observed by MLPA. Kit P250 (a) showed deletion of probes SNAP29, LZTR1 and HIC2 in Patient 1 and duplication of probes SNAP29 and LZTR1 in Patient 2, whereas kit P324-A1 (b) showed deletion of both LCRD probes in Patient 1 and duplication of only the proximal one in Patient 2. Data from both kits were analysed with Coffalyser v8 and v9, respectively.

However, MLPA testing of both patients and their parents with kits P023B, P250 and P324-A1 showed a deletion of 5 probes (SNAP29, LZTR1, LCRD probe 06317-L10754, HIC2 and LCRD probe 06321-L05796) on Patient 1 (Figure 2a, b), and a duplication of 3 probes (SNAP29, LZTR1 and LCRD probe 06317-L10754) on Patient 2 (Figure 2a, b) and his mother, uncle, two aunts and maternal grandfather (not shown). These MLPA loci fall within a shortest segment of 675.1 kb for the deletion and 207.4 kb for the duplication, both including the region between LCR22-C and LCR22-D [7]. On examination, the mother, uncle, two aunts and maternal grandfather of Patient 2 were all phenotypically normal.

On the other hand, microsatellite segregation studies were informative for STRs D22S311, D22S1709 and CATCH 38 in Patient 1, and confirmed a *de novo *paternal deletion. STR CATCH 42 showed a triallelic result for both Patient 2 and his mother, whereas CATCH 11 showed such a result only in the mother. Fluorescence intensity of the alleles of markers CATCH 41, D22S311 and CATCH 13 suggested a trisomic result (Table 1).

**Table 1:** Alleles of STR markers studied in Patient 1 and father and Patient 2 and relatives.

	Position	Patient 1	Father 1	Patient 2	Mother 2	Father 2	Maternal aunt 2a	Maternal aunt 2b	Maternal uncle 2	Maternal grandfather 2
CATCH 10	191,164,96	120 122	NA	120 122	122	120 122	122	122	122	122

CATCH 20	19,245,111	*160*	160	NA	160	160 162	160	160	160	160

PCQAP*	19,266,745	N	N	N	N	N	N	N	N	N

LCR-C**	19,354,000–19,417,000									

CATCH 42	19,449,222	*99*	93 99	**93 97 101**	**93 99 101**	97 99	**93 99 101**	**93 99 101**	**93 99 101**	**93 101**

CATCH 41	19,450,208	NA	169 173	**169 173**	**169 173**	173 177	**169 173**	**169 173**	**169 173**	**169 173**

CATCH 11	19,469,542	*197*	195 197	**195 199**	**191 195 199**	195 199	**191 195 199**	**191 195 199**	**191 195 199**	**195 199**

D22S311	19,503,575	**251**	249 255	**251 253**	**249 253**	251	**249 253**	**249 253**	**249 253**	**249 253**

CATCH 12	19,566,292	*116*	116	*116*	*116*	116	*116*	*116*	*116*	*116*

CATCH 13	19,567,154	*143*	138 143	**138 143**	**138 143**	143	**138 143**	**138 143**	**138 143**	**138 143**

SNAP29*	19,572,014	**del**	N	**dup**	**dup**	N	**dup**	**dup**	**dup**	**dup**

CATCH 14	19,595,454	*212*	212 215	**200 216 227**	**208 216 227**	200 210	**208 216 227**	**208 216 227**	**208 216 227**	**216 220 227**

CATCH 39	19,640,114	*106*	106 110	**94 102 110**	**94 102 106**	106 110	**94 102 106**	**94 102 106**	**94 102 106**	**94 102**

LZTR1*	19,679,191	**del**	N	**dup**	**dup**	N	**dup**	**dup**	**dup**	**dup**

D22S1709	19,735,440	**120**	128	**126 128 132**	**126 128 132**	128 132	**126 128 132**	**126 128 132**	**126 128 132**	**126 132**

CATCH 38	19,745,025	**157**	159	*159*	*159*	155 159	*159*	*159*	*159*	*159 163*

CATCH 37	19,759,833	NA	226 234	*230 245*	*230 245*	230 245	*230 245*	*230 245*	*230 245*	*230 245*

LCR-D**	19,777,000									

LCRD 06317-L10754*	19,779,435	**del**	N	**dup**	**dup**	N	**dup**	**dup**	**dup**	**dup**

CATCH 36	20,126,412	*109*	109	*109*	*109*	109	*109*	*109*	*109*	*109*

HIC2*	20,130,631	**del**	N	N	N	N	N	N	N	N

LCR-D**	20,242,000									

LCRD 06321-L05796*	20,247,101	**del**	N	N	N	N	N	N	N	N

CATCH 35	20,341,833	*136*	136 150	*136 150*	*136 143*	143 150	*136 143*	*136 143*	*136 143*	*136 143*

D22S446bis	20,343,665	*157*	157 185	157 180	157 161	165 180	157 161	157 161	157 161	157 161

PPIL2*	20,379,687	N	N	N	N	N	N	N	N	N

D22S306	20,887,523	*106*	106	NA	NA	NA	*106*	*106*	*106*	*106*

D22S303	21,599,366	212 222	212 222	NA	NA	NA	212 222	212 222	212 222	212

Alleles are expressed in fragment bp. STR markers are located according to the NCBI Build 36.1, together with the MLPA probes (*) and the LCRs (**). The shortest informative deletion and duplication segments by MLPA and STRs are D22S311-LCRD 06321-L05796 and CATCH 42-LCRD 06317-L10754, respectively. Bold text indicates haploinsufficiency or trisomy, two alleles listed in bold mean that homozygosity is suspected in one of them, so there are really three alleles. Regular text indicates biallelism. Italic indicates non informative results. del: deletion; dup: duplication; N: normal; NA: not available.

**Figure 3:**
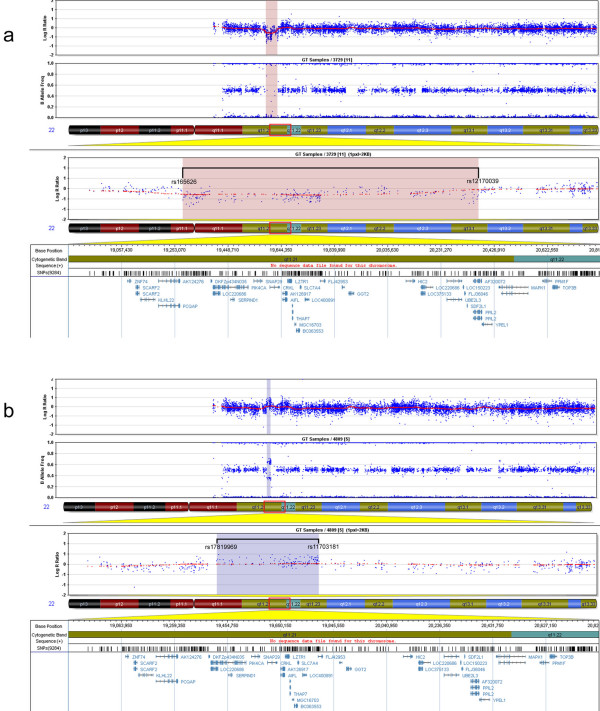
**Array-CGH SNP genotyping of Patients 1 and 2**. Chromosome 22 plot of Patients 1 (a) and 2 (b). The upper panels show the genic dosage (Log R ratio) and the homozygous/heterozygous distribution (B allele frequency) for all the SNPs genotyped. Both parameters are used together for interpretation of deletions and duplications. The region showing copy number alterations is zoomed in below, indicating the flanking SNPs and the genes in the region. A copy number decrease and loss of heterozygosity was found in Patient 1 (pink shading, a), whereas a copy number increase with four different allele populations was found in Patient 2 (blue shading, b).

**Figure 4:**
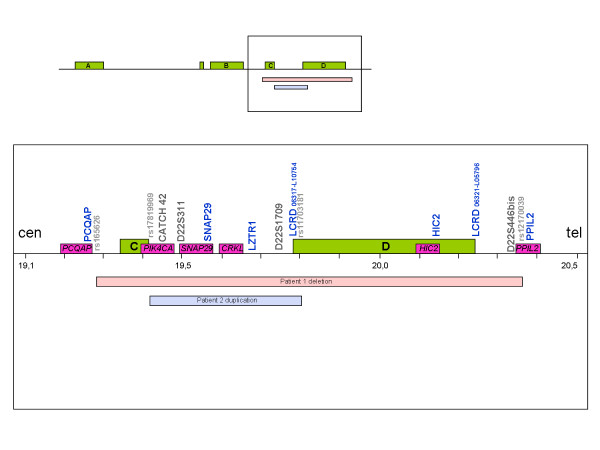
**Detailed map of the distal TDR in 22q11.2**. The relative location of the informative MLPA probes (blue font), STRs (black font) and SNPs (gray, small-sized font) sizing the shortest deletion and duplication size are shown above the line. Green boxes indicate LCRs, purple boxes indicate the discussed genes and the pink bar and the blue bar below the line indicate the deleted and the duplicated segment, respectively. Cen: centromere; tel: telomere.

SNP genotyping by array-CGH narrowed down the deletion size to 1092.027 kb (rs165626-rs12170039) in Patient 1 (Figure 3a) and the duplication size to 377.634 kb (rs17819969-rs11703181) for Patient 2 (Figure 3b). These results are consistent with MLPA and STRs studies. Other genomic copy number variations (CNV) observed in these patients had been previously described in the normal population [53,54] according to the Database of Genomic Variants [55] (data not shown). The relative location of each marker and the deleted and duplicated segments are shown in Figure 4.

## Discussion

The whole phenotypic spectrum of 22q11.2 deletion syndrome is contributed by multiple dosage-sensitive genes across the 22q11.2 region, that are required for normal development [reviewed in [1]]. Moreover, non-overlapping, atypical deletions do have significantly overlapping phenotypes, suggesting either, several candidate genes for the syndrome [13,14,56], a common developmental pathway [17] or a positional gene effect in 22q11.2 [17,57-59]. On the other hand, phenotypes shown by identical deletions in 22q11.2 have been suggested to be modified by parental imprinting [60], unbalanced regulatory effects [15,59], recessive mutations or polymorphisms unmasked by hemizygosity [59,60], environmental factors [60] or stochastic events during morphogenesis [15,61]. Susceptibility to other syndromes in patients with 22q11.2 deletions has also been proposed [58,62,63]. Phenotypic discordance has been observed in monozygotic twins with 22q11.2 deletion syndrome [33,64], a finding that is consistent with both a genetic and an environmental influence.

In the recent years, several candidate genes reproducing part of the phenotype in animal models [13,14,26,56,65] or suspected to change disease predisposition [66-69] have been identified in 22q11.2. In this context, correlations made within a contiguous gene syndrome will always be subjected to phenotype exceptions.

Rauch *et al *[19] described a likely genotype-phenotype correlation, by comparing the phenotypes of non-overlapping deletions in 22q11.2 (Table 2). They concluded that deletions covering the whole TDR or the proximally nested 1.5 Mb segment result in a phenotype characterized by conotruncal CHDs and typical VCFS, mainly by effects of haploinsufficiency of *TBX1*, a gene previously involved in the expression of these features when mutated [26], whereas distally nested deletions including *CRKL *would present with a milder, atypical phenotype with uncommon CHDs, developmental delay and mental impairment. Likewise, distal deletions which are adjacent to, and do not include the TDR, would generally have associated non-specific CHDs and very uncommon features of 22q11.2 deletion syndrome such as choanal atresia. Within these deletions, the shortest proximal segment has been associated with mild learning difficulties [19]. Recently, additional characteristic features of this new condition such as prematurity, prenatal and postnatal growth retardation, developmental delay, mild skeletal abnormalities and characteristic facial dysmorphic features have been described [23] (Table 2).

**Table 2:** Observed clinical features in atypical deletions of the LCR intervals in 22q11.2.

LCR interval	Rauch 2005 [19]		Ben-Shachar 2008 [23]	García-Miñaur 2002 [18]	Kurahashi 1996 [70]	D'Angelo 2007 [21]	Patient 1
	- ctCHD	- ctCHD					
A-B	- typical VCFS	- typical VCFS					
							
		- Atypical CHD		- ctCHD			
B-C		- Mild dysmorphism		- Mild facial dysmorphism			
		- DD		- Father normal			
					
		- Learning disabilities			- CTAFS: TOF, PA	- Mild facial features	- Atypical CHD
C-D					- Facial dysmorphism	- Neurodevelopmental, behavioural and psychiatric spectrum disorder	- Atypical facial features
						- Mother with depressive disorder	

			- Prematurity				
	- Mild DD	- Mild DD					
			- Prenatal and postnatal GR				
	- Choanal atresia						
			- Learning difficulties and/or DD				
D-E	- Atypical CHD		- Specific skeletal abnormalities				
			- Characteristic facial dysmorphism				
			- Higher risk for TA				
							
E-F							

CTAFS: conotruncal anomaly face syndrome; ctCHD: conotruncal congenital heart defect; DD: developmental delay; GR: growth retardation; PA: pulmonary atresia; TA: truncus arteriosus; TOF: tetralogy of Fallot; VCFS: Velocardiofacial syndrome.

The absence of a genotype-phenotype correlation highlights the incomplete penetrance associated with alterations in the 22q11.2 region. As an example, an apparently identical atypical deletion in two reported familial cases was found to present both inter- and intrafamilial variability [18,19]. This atypical deletion which spanned the B-D segment, and which should have been mainly involved in learning disability and mental retardation, was found to be associated with a conotruncal CHD in a child of a deleted father with normal phenotype [18] (Table 2). Whereas, in the second case, the child developed a more neurobehavioural phenotype, with an undiagnosed father with schizophrenia [19]. Both patients had mild facial dysmorphism with overlapping features of DGS/VCFS.

In line with this, the only two reported distally nested C-D deletions similar to that found in Patient 1 are also phenotypically discordant [21,70]. Both were located within this segment that should be associated with the neurobehavioural expression. The patient from D'Angelo *et al *[21] had mild facial features and a complex neurodevelopmental, behavioural and psychiatric spectrum, with a major depressive disorder in her transmitting mother, but the patient described by Kurahashi *et al *[70] had a typical conotruncal anomaly face syndrome with pulmonary atresia, tetralogy of Fallot, and facial dysmorphic features. Our patient had a non-specific CHD and mild facial features, barely suggestive of the 22q11.2 deletion syndrome (Table 2). All these findings, within such a small sample size, do not reveal strong evidences but suggest that genetic changes in 22q11.2 are incompletely penetrant [19] or that maybe genotype-phenotype correlations are invalid for this specific region [21].

The reason for all these exceptions in the distal TDR might be the presence of *CRKL*, a gene that has been involved in multisystemic affection of cardiac neural crest derivatives when null-mutated in mice [65]. Otherwise, other genes in this region seem to be responsible for a more particular phenotype: certain SNP haplotypes within genes *PIK4CA *[66,67] and *SNAP29 *[68,69] have been recently suggested to confer a higher risk of developing schizophrenia (Figure 4).

Recently, high resolution molecular diagnostic techniques have contributed to map the rearrangements in 22q11.2 more precisely. In fact, high resolution comparative genomic hybridization (HR-CGH), real-time quantitative PCR (qPCR) and high density MLPA (HD-MLPA) have shown that around 7% of the typical 3 Mb A-D deletions have atypical distal breakpoints covering *HIC2*, suggesting that this gene located within LCR22-D might be involved in the phenotypic variability among these patients [20,71,72]. *Hic2 *may be required for heart development in mouse [Lammerts van Beuren K and Scambler PJ, personal communication]. *HIC2 *might be also involved in the phenotypic variability of Patient 1 and other patients reported to carry the C-D deletion [21,70], as well as in patients with other atypical deletions [15,17,19,20,58].

Screening for duplications at 22q11.2 has no standardized procedure, as these patients show clinical manifestations, only some of which are compatible with 22q11.2 deletion syndrome within a generally milder phenotype [37,41,43,48]. Different duplication sizes have been reported [43,48,49], all of them showing a higher tendency to be inherited than deletions [43]. Phenotype correlations to the sizes and locations of these duplications are even harder to find than in deletions, as the whole clinical spectrum is still unknown and many of these individuals overlap normality [41,43].

Patient 2 and his maternal relatives are carriers of an atypical duplication that maps distal within the TDR. Unlike common duplications [43,63,73], this novel rearrangement, the smallest one reported to date, has therefore unknown clinical implications. Polymorphisms and copy number variants can be defined as alterations of a small region known to contain no critical genes and that can be inherited from healthy individuals [41,43]. However, as proposed for *TBX1*, other genes in 22q11.2 may have clinical significance when present in a trisomic dosage [43,74,75]. On the other hand, four other carriers of the family of Patient 2 show normal phenotype, and the patient shows cleft palate, a potentially multifactorial malformation which may be less associated with 22q11.2 duplications than thought [40]. The case that is most similar to this duplication was described by Fan *et al *[76] in a patient with mental retardation or developmental delay associated to other malformations, inherited from a phenotypically normal father. This 479 kb duplication also included *PCQAP*, another gene with a potential involvement in schizophrenia susceptibility [77,78].

Novel and rare rearrangements in such a complex chromosomal region with highly uncertain phenotypic consequences, have uncertain implications for genetic counseling. High intrafamilial variability and reduced penetrance are phenotypic effects that hamper the distinction between pathologic change and polymorphism [79]. In this context, the possibility that the phenotypes of our patients might not be caused by the observed rearrangements should be reminded. Also, small deletions and duplications occurring from generation to generation in this rearranged segment cannot be excluded [80].

Despite having occurred in the same region, our two rearrangements, as far as they have been molecularly characterized, are significantly different (Figure 4). The deletion is more than twice the size of the duplication and includes *HIC2 *and the whole LCRs C and D, which are nearly intact in the duplication. This variation in the breakpoints location highlights potential differences in the molecular mechanisms mediating these rearrangements. In fact, the deletion is too large to be a C-D rearrangement and is possibly not mediated by non-allelic homologous recombination between LCRs.

Even for identical rearrangements, genetic counseling and clinical prognosis is still complex. Genotype-phenotype correlations made so far in 22q11.2 are compatible with a clinical outcome modified by environmental/stochastic events [15,60,61] and eclipsed by regulatory factors [15,59]. At this moment, the molecular diagnostic techniques we have used are the best screening procedure for patients with this whole spectrum of overlapping clinical features, suspected of having any kind of rearrangement in 22q11.2 resulting in a copy number imbalance [71,81,82].

## Conclusion

In summary, we report on the third atypical deletion and the first atypical duplication in the vicinity of low-copy repeats C and D in distal 22q11.2 deletion region. The breakpoints location and sizes make it possible that both rearrangements may be mediated by different mechanisms or sequences. A complex network of modifying factors may influence the phenotypic outcome of these chromosomal aberrations. A systematic literature review and genotyping of phenotype-associated polymorphisms can help anticipating the clinical evolution of patients and understanding the prognosis and predisposition to this disease.

## List of abbreviations used

Array-CGH: Array-based comparative genomic hybridization; ASD: Atrial septal defect; CES: Cat Eye syndrome; CHD: Congenital heart defect; CNV: Copy number variation; DGS: DiGeorge syndrome; FISH: Fluorescence *in situ *hybridization; HD-MLPA: High density multiplex ligation-dependent probe amplification; HR-CGH: High resolution comparative genomic hybridization; IRB: Institutional Review Board; LCR: Low-copy repeat; MLPA: Multiplex ligation-dependent probe amplification; NAHR: Non-allelic homologous recombination; OFC: Occipitofrontal circumference; qPCR: Quantitative polymerase chain reaction; SNP: Single nucleotide polymorphism; SRO: Shortest region of overlap; TDR: Typically deleted region; VCFS: Velocardiofacial syndrome; VSD: Ventricular septal defect.

## Competing interests

The authors declare that they have no competing interests.

## Authors' contributions

LF carried out the molecular genetic studies and wrote the manuscript. JN carried out the molecular genetic studies and participated in the draft of the manuscript. FS carried out the clinical genetic characterization of Patient 2. DHS carried out the microsatellites design and participated in the draft of the manuscript. VMG carried out the SNP array and CNV data analysis and interpretation. SGM and RP carried out the clinical genetic characterization of the family of Patient 2. AD, ILP and MP carried out the cytogenetic and FISH studies. LGG carried out the cardiological evaluation and follow-up of Patient 1. EV and FH carried out the clinical evaluation and follow-up of Patient 2. PL conceived the study, carried out the clinical genetic characterization of Patients 1 and 2 and participated in the draft of the manuscript. All authors read and approved the final manuscript.

## Pre-publication history

The pre-publication history for this paper can be accessed here:



## References

[B1] Lindsay EA (2001). Chromosomal microdeletions: dissecting del22q11 syndrome. Nat Rev Genet.

[B2] Ryan AK, Goodship JA, Wilson DI, Philip N, Levy A, Seidel H, Schuffenhauer, Oechsler H, Belohradsky B, Prieur M, Aurias A, Raymond FL, Clayton-Smith J, Hatchwell E, McKeown C, Beemer FA, Dallapiccola B, Novelli G, Hurst JA, Ignatius J, Green AJ, Winter RM, Brueton L, Brondum-Nielsen K, Stewart F, Van Essen T, Patton M, Paterson J, Scambler PJ (1997). Spectrum of clinical features associated with interstitial chromosome 22q11 deletions: a European collaborative study. J Med Genet.

[B3] Robin NH, Shprintzen RJ (2005). Defining the clinical spectrum of deletion 22q11.2. J Pediatr.

[B4] Carey AH, Kelly D, Halford S, Wadey R, Wilson D, Goodship J, Burn J, Paul T, Sharkey A, Dumanski J, Nordenskjold M, Williamson R, Scambler PJ (1992). Molecular genetic study of the frequency of monosomy 22q11 in DiGeorge syndrome. Am J Hum Genet.

[B5] Carlson C, Sirotkin H, Pandita R, Goldberg R, McKie J, Wadey R, Patanjali SR, Weissman SM, Anyane-Yeboa K, Warburton D, Scambler P, Shprintzen R, Kucherlapati R, Morrow BE (1997). Molecular definition of 22q11 deletions in 151 velo-cardio-facial syndrome patients. Am J Hum Genet.

[B6] Thomas JA, Graham JM (1997). Chromosome 22q11 deletion syndrome: an update and review for the primary pediatrician. Clin Pediatr.

[B7] Shaikh TH, Kurahashi H, Saitta SC, O'Hare AM, Hu P, Roe BA, Driscoll DA, McDonald-McGinn DM, Zackai EH, Budarf ML, Emanuel BS (2000). Chromosome 22-specific low copy repeats and the 22q11.2 deletion syndrome: genomic organization and deletion endpoint analysis. Hum Mol Genet.

[B8] Saitta SC, Harris SE, Gaeth AP, Driscoll DA, McDonald-McGinn DM, Maisenbacher MK, Yersak JM, Chakraborty PK, Hacker AM, Zackai EH, Ashley T, Emanuel BS (2004). Aberrant interchromosomal exchanges are the predominant cause of the 22q11.2 deletion. Hum Mol Genet.

[B9] Baumer A, Riegel M, Schinzel A (2004). Non-random asynchronous replication in 22q11.2 favours unequal meiotic crossovers leading to the human 22q11.2 deletion. J Med Genet.

[B10] Shaikh TH, O'Connor RJ, Pierpont ME, McGrath J, Hacker AM, Nimmakayalu M, Geiger E, Emanuel BS, Saitta SC (2007). Low copy repeats mediate distal chromosome 22q11.2 deletions: sequence analysis predicts breakpoint mechanisms. Genome Res.

[B11] Kurahashi H, Tsuda E, Kohama R, Nakayama T, Masuno M, Imaizumi K, Kamiya T, Sano T, Okada S, Nishisho I (1997). Another critical region for deletion of 22q11: a study of 100 patients. Am J Med Genet.

[B12] O'Donnell H, McKeown C, Gould C, Morrow B, Scambler P (1997). Detection of an atypical 22q11 deletion that has no overlap with the DiGeorge syndrome critical region. Am J Hum Genet.

[B13] Yamagishi H, Garg V, Matsuoka R, Thomas T, Srivastava D (1999). A molecular pathway revealing a genetic basis for human cardiac and craniofacial defects. Science.

[B14] McQuade L, Christodoulou J, Budarf M, Sachdev R, Wilson M, Emanuel B, Colley A (1999). Patient with a 22q11.2 deletion with no overlap of the minimal DiGeorge syndrome critical region (MDGCR). Am J Med Genet.

[B15] Amati F, Conti E, Novelli A, Bengala M, Digilio MC, Marino B, Giannotti A, Gabrielli O, Novelli G, Dallapiccola B (1999). Atypical deletions suggest five 22q11.2 critical regions related to the DiGeorge/velo-cardio-facial syndrome. Eur J Hum Genet.

[B16] Lu JH, Chung MY, Betau H, Chien HP, Lu JK (2001). Molecular characterization of tetralogy of fallot within DiGeorge critical region of the chromosome 22. Pediatr Cardiol.

[B17] Saitta SC, McGrath JM, Mensch H, Shaikh H, Zackai EH, Emanuel BS (1999). A 22q11.2 deletion that excludes *UFD1L *and *CDC45L *in a patient with conotruncal and craniofacial defects. Am J Hum Genet.

[B18] Garcia-Miñaur S, Fantes J, Murray RS, Porteous MEM, Strain L, Burns JE, Stephen J, Warner JP (2002). A novel atypical 22q11.2 distal deletion in father and son. J Med Genet.

[B19] Rauch A, Zink S, Zweier C, Thiel T, Koch A, Rauch R, Lascorz J, Hüffmeier U, Weyand M, Singer H, Hofbeck M (2005). Systematic assessment of atypical deletions reveals genotype-phenotype correlation in 22q11.2. J Med Genet.

[B20] Weksberg R, Stachon AC, Squire JA, Moldovan L, Bayani J, Meyn S, Chow E, Bassett AS (2007). Molecular characterization of deletion breakpoints in adults with 22q11 deletion syndrome. Hum Genet.

[B21] D'Angelo CS, Jehee FS, Koiffmann CP (2007). An inherited atypical 1 Mb 22q11.2 deletion within the DGS/VCFS 3 Mb region in a child with obesity and aggressive behavior. Am J Med Genet A.

[B22] Nogueira SI, Hacker AM, Bellucco FT, Christofolini DM, Kulikowski LD, Cernach MC, Emanuel BS, Melarango MI (2008). Atypical 22q11.2 deletion in a patient with DGS/VCFS spectrum. Eur J Med Genet.

[B23] Ben-Shachar S, Ou Z, Shaw CA, Belmont JW, Patel MS, Hummel M, Amato S, Tartaglia N, Berg J, Sutton VR, Lalani SR, Chinault AC, Cheung SW, Lupski JR, Patel A (2008). 22q11.2 distal deletion: a recurrent genomic disorder distinct from DiGeorge syndrome and velocardiofacial syndrome. Am J Hum Genet.

[B24] Uddin RK, Zhang Y, Siu VM, Fan YS, O'Reilly RL, Rao J, Singh SM (2006). Breakpoint associated with a novel 2.3 Mb deletion in the VCFS region of 22q11 and the role of Alu (SINE) in recurring microdeletions. BMC Med Genet.

[B25] Gong W, Gottlieb S, Collins J, Blescia A, Dietz H, Goldmuntz E, McDonald-McGinn DM, Zackai EH, Emanuel BS, Driscoll DA, Budarf ML (2001). Mutation analysis of *TBX1 *in non-deleted patients with features of DGS/VCFS or isolated cardiovascular defects. J Med Genet.

[B26] Yagi H, Furutani Y, Hamada H, Sasaki T, Asakawa S, Minoshima S, Ichida F, Joo K, Kimura M, Imamura S, Kamatani N, Momma K, Takao A, Nakazawa M, Shimizu N, Matsuoka R (2003). Role of *TBX1 *in human del22q11.2 syndrome. Lancet.

[B27] Augusseau S, Jouk S, Jalbert P, Prieur M (1986). DiGeorge syndrome and 22q11 rearrangements. Hum Genet.

[B28] Lindsay EA, Halford S, Wadey R, Scambler PJ, Baldini A (1993). Molecular cytogenetic characterization of the DiGeorge syndrome region using fluorescence in situ hybridization. Genomics.

[B29] Holmes SE, Riazi MA, Gong W, McDermid HE, Sellinger BT, Hua A, Chen F, Wang Z, Zhang G, Roe B, Gonzalez I, McDonald-McGinn DM, Zackai E, Emanuel BS, Budarf ML (1997). Disruption of the clathrin heavy chain-like gene (CLTCL) associated with features of DGS/VCFS: a balanced (21;22)(p12;q11) translocation. Hum Mol Genet.

[B30] Halford S, Wadey R, Roberts C, Daw SC, Whiting JA, O'Donnell H, Dunham I, Bentley D, Lindsay E, Baldini A, Francis F, Lehrach H, Williamson R, Wilson DI, Goodship J, Cross I, Burn J, Scambler PJ (1993). Isolation of a putative transcriptional regulator from the region of 22q11 deleted in DiGeorge syndrome, Shprintzen syndrome and familial congenital heard disease. Hum Mol Genet.

[B31] Novelli G, Amati F, Dallapiccola B (1999). UFD1L and CDC45L: a role in DiGeorge syndrome and related phenotypes?. Trends Genet.

[B32] Lindsay EA, Goldberg R, Jurecic V, Morrow B, Carlson C, Kucherlapati RS, Shprintzen RJ, Baldini A (1995). Velo-cardio-facial syndrome: frequency and extent of 22q11 deletions. Am J Med Genet.

[B33] Yamagishi H, Ishii C, Maeda J, Kojima Y, Matsuoka R, Kimura M, Takao A, Momma K, Matsuo N (1998). Phenotypic discordance in monozygotic twins with 22q11.2 deletion. Am J Med Genet.

[B34] McDonald-McGinn DM, Tonnesen MK, Laufer-Cahana A, Finucane B, Driscoll DA, Emanuel BS, Zackai EH (2001). Phenotype of the 22q11.2 deletion in individuals identified through an affected relative: cast a wide FISHing net!. Genet Med.

[B35] Fernández L, Lapunzina P, Pajares IL, Criado GR, García-Guereta L, Pérez J, Quero J, Delicado A (2005). Higher frequency of uncommon 1.5–2 Mb deletions found in familial cases of 22q11.2 deletion syndrome. Am J Med Genet.

[B36] Mikhail FM, Descartes M, Piotrowski A, Andersson R, de Ståhl TD, Komorowski J, Bruder CE, Dumanski JP, Carroll AJ (2007). A previously unrecognized microdeletion syndrome on chromosome 22 band q11.2 encompassing the *BCR *gene. Am J Med Genet A.

[B37] Ensenauer RE, Adeyinka A, Flynn HC, Michels VV, Lindor NM, Dawson DB, Thorland EC, Lorentz CP, Goldstein JL, McDonald MT, Smith WE, Simon-Fayard E, Alexander AA, Kulharya AS, Ketterling RP, Clark RD, Jalal SM (2003). Microduplication 22q11.2, an emerging síndrome: clinical, cytogenetic and molecular analysis of thirteen patients. Am J Hum Genet.

[B38] Portnoï MF, Lebas F, Gruchy N, Ardalan A, Biran-Mucignat V, Malan V, Finkel L, Roger G, Ducrocq S, Gold F, Taillemite JL, Marlin S (2005). 22q11.2 duplication syndrome: two new familial cases with some overlapping features with DiGeorge/velocardiofacial syndromes. Am J Med Genet A.

[B39] Brunet A, Gabau E, Perich RM, Valdesoiro L, Brun C, Caballín MR, Guitart M (2006). Microdeletion and microduplication 22q11.2 screening in 295 patients with clinical features of DiGeorge/velocardiofacial syndrome. Am J Med Genet A.

[B40] Sivertsen Ǻ, Lie RT, Wilcox AJ, Ǻbyholm F, Vindenes H, Haukanes BI, Houge G (2007). Prevalence of duplications and deletions of the 22q11 DiGeorge syndrome region in a population-based sample of infants with cleft palate. Am J Med Genet A.

[B41] Courtens W, Schramme I, Laridon A (2008). Microduplication 22q11.2: a benign polymorphism or a syndrome with a very large clinical variability and reduced penetrance? – Report of two families. Am J Med Genet A.

[B42] Dempsey MA, Schwartz S, Waggoner DJ (2007). Mosaicism del(22)(q11.2q11.2)/dup(22)(q11.2q11.2) in a patient with features of 22q11.2 deletion syndrome. Am J Med Genet A.

[B43] Ou Z, Berg JS, Yonath H, Enciso VB, Miller DT, Picker J, Lenzi T, Keegan CE, Sutton VR, Belmont J, Chinault AC, Lupski JR, Cheung SW, Roeder E, Patel A (2008). Microduplications of 22q11.2 are frequently inherited and are associated with variable phenotypes. Genet Med.

[B44] Edelmann L, Pandita RK, Spiteri E, Funke B, Goldberg R, Palanisamy N, Chaganti RS, Magenis E, Shprintzen RJ, Morrow BE (1999). A common molecular basis for rearrangement disorders on chromosome 22q11. Hum Mol Genet.

[B45] Yobb TM, Somerville MJ, Willatt L, Firth HV, Harrison K, MacKenzie J, Gallo N, Morrow BE, Shaffer LG, Babcock M, Chernos J, Bernier F, Sprysak K, Christiansen J, Haase S, Elyas B, Lilley M, Bamforth S, McDermid HE (2005). Microduplication and triplication of 22q11.2: a highly variable syndrome. Am J Hum Genet.

[B46] Yu S, Cox K, Friend K, Smith S, Buchheim R, Bain S, Liebelt J, Thompson E, Bratkovic D (2008). Familial 22q11.2 duplication: a three-generation family with a 3-Mb duplication and a familial 1.5-Mb duplication. Clin Genet.

[B47] De la Rochebrochard C, Joly-Hélas G, Goldenberg A, Durand I, Laquerrière A, Ickowicz V, Saugier-Veber P, Eurin D, Moirot H, Diguet A, de Kergal F, Tiercin C, Mace B, Marpeau L, Frebourg T (2006). The intrafamilial variability of the 22q11.2 microduplication encompasses a spectrum from minor cognitive deficits to severe congenital anomalies. Am J Med Genet A.

[B48] Wentzel C, Fernström M, Öhrner Y, Annerén G, Thuresson AC (2008). Clinical variability of the 22q11.2 duplication syndrome. Eur J Med Genet.

[B49] Alberti A, Romano C, Falco M, Calì F, Schinocca P, Galesi O, Spalletta A, Di Benedetto D, Fichera M (2007). 1.5 Mb de novo 22q11.21 microduplication in a patient with cognitive deficits and dysmorphic facial features. Clin Genet.

[B50] MRC-Holland: Multiplex Ligation-dependent Probe Amplification. http://www.mlpa.com/WebForms/WebFormMain.aspx?Tag=fNPBLedDVp38p\CxU2h0mQ||.

[B51] Torres-Juan L, Rosell J, Sánchez-de-la-Torre M, Fibla J, Heine-Suñer D (2007). Analysis of meiotic recombination of 22q11.2, a region that frequently undergoes deletions and duplications. BMC Med Genet.

[B52] Simon-Sanchez J, Scholz S, Fung HC, Matarin M, Hernandez D, Gibbs JR, Britton A, de Vrieze FW, Peckham E, Gwinn-Hardy K, Crawley A, Keen JC, Nash J, Borgaonkar D, Hardy J, Singleton A (2007). Genome-wide SNP assay reveals structural genomic variation, extended homozygosity and cell-line induced alterations in normal individuals. Hum Mol Genet.

[B53] Pinto D, Marshall C, Feuk L, Scherer SW (2008). Copy-number variation in control population cohorts. Hum Mol Genet.

[B54] Perry GH, Ben-Dor A, Tsalenko A, Sampas N, Rodriguez-Revenga L, Tran CW, Scheffer A, Steinfeld I, Tsang P, Yamada NA, Park HS, Kim JI, Seo JS, Yakhini Z, Laderman S, Bruhn L, Lee C (2008). The fine-scale and complex architecture of human copy-number variation. Am J Hum Genet.

[B55] Database of Genomic Variants. A curated catalogue of structural variation in the human genome. http://projects.tcag.ca/variation/?source=hg18.

[B56] Guris DL, Fantes J, Tara D, Druker BJ, Imamoto A (2001). Mice lacking the homologue of the human 22q11.2 gene CRKL phenocopy neurocristopathies of DiGeorge syndrome. Nat Genet.

[B57] Sutherland HF, Wadey R, McKie JM, Taylor C, Atif U, Johnstone KA, Halford S, Kim UJ, Goodship J, Baldini A, Scambler PJ (1996). Identification of a novel transcript disrupted by a balanced translocation associated with DiGeorge syndrome. Am J Hum Genet.

[B58] Rauch A, Pfeiffer RA, Leipold G, Singer H, Tigges M, Hofbeck M (1999). A novel 22q11.2 microdeletion in DiGeorge syndrome. Am J Hum Genet.

[B59] Dallapiccola B, Pizzuti A, Novelli G (1996). How many breaks do we need to CATCH on 22q11?. Am J Hum Genet.

[B60] Hall GJ (1993). CATCH 22. J Med Genet.

[B61] Kurnit DM, Layton WM, Matthysse S (1987). Genetics, chance, and morphogenesis. Am J Hum Genet.

[B62] Digilio MC, Marino B, Capolino R, Angioni A, Sarkozy A, Roberti MC, Conti E, de Zorzi A, Dallapiccola B (2005). Familial recurrence of nonsyndromic congenital heart defects in first degree relatives of patients with deletion 22q11.2. Am J Med Genet.

[B63] Heine-Suñer D, Armengol L, Torres-Juan L, de la Fuente M, García-Algas F, Fernández L, Reyero M, Juan M, Tubau A, Pérez-Granero A, Bernués M, Govea N, Lapunzina P, Estivill X, Rosell J (2008). Clinical and molecular characterization of deletions, duplications and mutations in the 22q11.2 region [abstract]. Eur J Hum Genet.

[B64] Goodship J, Cross I, Scambler P, Burn J (1995). Monozygotic twins with chromosome 22q11 deletion and discordant phenotype. J Med Genet.

[B65] Guris DL, Fantes J, Tara D, Druker BJ, Imamoto A (2001). Mice lacking the homologue of the human 22q11.2 gene CRKL phenocopy neurocristopathies of DiGeorge syndrome. Nat Genet.

[B66] Jungerius BJ, Hoogendoorn ML, Bakker SC, Van't Slot R, Bardoel AF, Ophoff RA, Wijmenga C, Kahn RS, Sinke RJ (2008). An association screen of myelin-related genes implicates the chromosome 22q11 PIK4CA gene in schizophrenia. Mol Psychiatry.

[B67] Vorstman JAS, Chow EW, Ophoff RA, van Engeland H, Beemer FA, Kahn RS, Sinke RJ, Bassett AS (2009). Association of the PIK4CA schizophrenia-susceptibility gene in adults with the 22q11.2 deletion syndrome. Am J Med Genet B Neuropsychiatr Genet.

[B68] Saito T, Guan F, Papolos DF, Rajouria N, Fann CS, Lachman HM (2001). Polymorphism in SNAP29 gene promoter region associated with schizophrenia. Mol Psychiatry.

[B69] Wonodi I, Hong LE, Avila MT, Buchanan RW, Carpenter WT, Stine OC, Mitchell BD, Thaker GK (2005). Association between polymorphism of the SNAP29 gene promoter region and schizophrenia. Schizophr Res.

[B70] Kurahashi H, Nakayama T, Osugi Y, Tsuda E, Masuno M, Imaizumi K, Kamiya T, Sano T, Okada S, Nishisho I (1996). Deletion mapping of 22q11 in CATCH22 syndrome: identification of a second critical region. Am J Hum Genet.

[B71] Jalali GR, Vorstman JA, Errami A, Vijzelaar R, Biegel J, Shaikh T, Emanuel BS (2008). Detailed analysis of 22q11.2 with a high density MLPA probe set. Hum Mutat.

[B72] Urban AE, Korbel JO, Selzer R, Richmond T, Hacker A, Popescu GV, Cubells JF, Green R, Emanuel BS, Gernstein MB, Weissman SM, Snyder M (2006). High-resolution mapping of DNA copy alterations in human chromosome 22 using high-density tiling oligonucleotide arrays. Proc Natl Acad Sci USA.

[B73] Blennow E, Lagerstedt K, Malmgren H, Sahlén S, Schoumans J, Anderlid B (2008). Concurrent microdeletion and duplication of 22q11.2. Clin Genet.

[B74] Zweier C, Sticht H, Aydin-Yaylagül I, Campbell CE, Rauch A (2007). Human TBX1 missense mutations cause gain of function resulting in the same phenotype as 22q11.2 deletions. Am J Hum Genet.

[B75] Torres-Juan L, Rosell J, Morla M, Vidal-Pou C, García-Algas F, de la Fuente MA, Juan M, Tubau A, Bachiller D, Bernues M, Perez-Granero A, Govea N, Busquets X, Heine-Suñer D (2007). Mutations in TBX1 genocopy the 22q11.2 deletion and duplication syndromes: a new susceptibility factor for mental retardation. Eur J Hum Genet.

[B76] Fan YS, Jayakar P, Zhu H, Barbouth D, Sacharow S, Morales A, Carver V, Benke P, Mundy P, Elsas LJ (2007). Detection of pathogenic gene copy number variations in patients with mental retardation by genomewide oligonucleotide array comparative genomic hybridization. Hum Mutat.

[B77] De Luca A, Conti E, Grifone N, Amati F, Spalletta G, Caltagirone C, Bonaviri G, Pasini A, Gennarelli M, Stefano B, Berti L, Mittler G (2003). Association study between CAG trinucleotide repeats in the PCQAP gene (PC2 glutamine/Q-rich-associated protein) and schizophrenia. Am J Med Genet B Neuropsychiatr Genet.

[B78] Sandhu HK, Hollenbeck N, Wassink TH, Philibert RA (2004). An association study of PCQAP polymorphisms and schizophrenia. Psychiatr Genet.

[B79] Mefford H, Sharp A, Baker C, Itsara A, Jiang Z, Buysse K, Huang S, Maloney V, Crolla J, Baralle D, Collins A, Mercer C, Norga K, de Ravel T, Devriendt K, Bongers E, de Leeuw N, Reardon W, Gimelli S, Bena F, Hennekam R, Male A, Gaunt L, Clayton-Smith J, Simonic I, Park S, Mehta S, Nik-Zainal S, Woods C, Firth H, Parkin G, Fichera M, Reitano S, Lo Giudice M, Li K, Casuga I, Broomer A, Conrad B, Schwerzmann M, Räber L, Gallati S, Striano P, Coppola A, Tolmie J, Tobias E, Lilley C, Armengol L, Spysschaert Y, Verloo P, De Coene A, Goosens L, Mortier G, Speleman F, van Binsbergen E, Nelen M, Hochstenbach R, Poot M, Gallagher L, Gill M, McClellan J, King MC, Regan R, Skinner C, Stevenson R, Antonarakis S, Chen C, Estivill X, Menten B, Gimelli G, Gribble S, Schwartz S, Sutcliffe J, Walsh T, Knight S, Sebat J, Romano C, Schwartz C, Veltman J, de Vries B, Vermeesch J, Barber J, Willatt L, Tassabehji M, Eichler E (2008). Recurrent rearrangements of chromosome 1q21.1 and variable pediatric phenotypes. N Engl J Med.

[B80] Wieser R, Fritz B, Ullmann R, Müller I, Galhuber M, Storlazzi CT, Ramaswamy A, Christiansen H, Shimizu N, Rehder H (2005). Novel rearrangement of chromosome band 22q11.2 causing 22q11.2 microdeletion syndrome-like phenotype and rhabdoid tumor of the kidney. Hum Mutat.

[B81] Fernández L, Lapunzina P, Arjona D, López Pajares I, García-Guereta L, Elorza D, Burgueros M, De Torres ML, Mori MA, Palomares M, García-Alix A, Delicado A (2005). Comparative study of three diagnostic approaches (FISH, STRs and MLPA) in 30 patients with 22q11.2 deletion syndrome. Clin Genet.

[B82] Vorstman JA, Jalali GR, Rappaport EF, Hacker AM, Scott C, Emanuel BS (2006). MLPA: a rapid, reliable, and sensitive method for detection and analysis of abnormalities of 22q. Hum Mutat.

